# Consensus genomic regions associated with grain protein content in hexaploid and tetraploid wheat

**DOI:** 10.3389/fgene.2022.1021180

**Published:** 2022-09-28

**Authors:** Pooja Saini, Imran Sheikh, Dinesh Kumar Saini, Reyazul Rouf Mir, Harcharan Singh Dhaliwal, Vikrant Tyagi

**Affiliations:** ^1^ Department of Genetics-Plant Breeding and Biotechnology, Dr. Khem Singh Gill Akal College of Agriculture, Eternal University, Baru Sahib, India; ^2^ Department of Plant Breeding and Genetics, Punajb Agricultural University, Ludhiana, India; ^3^ Division of Genetics and Plant Breeding, Faculty of Agriculture SKUAST-Kashmir, Srinagar, India

**Keywords:** mQTLs, QTL hotspots, candidate gene, expression analysis, wheat

## Abstract

A meta-analysis of QTLs associated with grain protein content (GPC) was conducted in hexaploid and tetraploid wheat to identify robust and stable meta-QTLs (MQTLs). For this purpose, as many as 459 GPC-related QTLs retrieved from 48 linkage-based QTL mapping studies were projected onto the newly developed wheat consensus map. The analysis resulted in the prediction of 57 MQTLs and 7 QTL hotspots located on all wheat chromosomes (except chromosomes 1D and 4D) and the average confidence interval reduced 2.71-fold in the MQTLs and QTL hotspots compared to the initial QTLs. The physical regions occupied by the MQTLs ranged from 140 bp to 224.02 Mb with an average of 15.2 Mb, whereas the physical regions occupied by QTL hotspots ranged from 1.81 Mb to 36.03 Mb with a mean of 8.82 Mb. Nineteen MQTLs and two QTL hotspots were also found to be co-localized with 45 significant SNPs identified in 16 previously published genome-wide association studies in wheat. Candidate gene (CG) investigation within some selected MQTLs led to the identification of 705 gene models which also included 96 high-confidence CGs showing significant expressions in different grain-related tissues and having probable roles in GPC regulation. These significantly expressed CGs mainly involved the genes/gene families encoding for the following proteins: aminotransferases, early nodulin 93, glutamine synthetases, invertase/pectin methylesterase inhibitors, protein BIG GRAIN 1-like, cytochrome P450, glycosyl transferases, hexokinases, small GTPases, UDP-glucuronosyl/UDP-glucosyltransferases, and EamA, SANT/Myb, GNAT, thioredoxin, phytocyanin, and homeobox domains containing proteins. Further, eight genes including *GPC-B1, Glu-B1-1b, Glu-1By9, TaBiP1*, *GSr*, *TaNAC019-A*, *TaNAC019-D*, and *bZIP-TF SPA* already known to be associated with GPC were also detected within some of the MQTL regions confirming the efficacy of MQTLs predicted during the current study.

## Introduction

The hexaploid bread wheat (*Triticum aestivum* L.) is the major food crop for approximately one-third of the world population with 760.93 million thousand tonnes of production from a growing area of over 219 million thousand hectares (https://www.fao.org). It constitutes the 20 percent dietary component of both calories and protein in the human diet (Peng et al., 2011; Pal et al., 2022). The tetraploid durum wheat (*Triticum turgidum* L. subsp. Durum Desf.) is mainly used for pasta making. The most extensively produced species is common wheat (95%) followed by durum wheat accounting for the remaining 5%. Given the ever-increasing emphasis on health among consumers, wheat breeding efforts have recently shifted their focus from enhancing production to enriching quality end products with high nutritional value (Saini et al., 2020). Wheat quality is a versatile and complex phenomenon involving various factors (Peng et al., 2022). Both protein content, as well as the quality of processed wheat products, is primarily governed by grain protein content (GPC) and protein quality (protein profile). Wheat proteins are challenging to define due to their tremendous complexity in genetic factors and diverse environmental influence with one another.

Wheat grain storage proteins are a complex mixture of various polypeptide chains that have typically been categorized based on their solubility or composition and structure (Peng et al., 2022). GPC has an important role in determining the crop’s commercial worth by altering the end-use quality and nutritional content of flour/semolina. Given that mature wheat grains typically contain 8–16% protein (Žilić et al., 2011), one of the breeders’ key goals is to find stable QTLs and superior alleles that can be successfully introgressed from high GPC lines to low GPC lines but superior in terms of agronomic traits (Kumar et al., 2018). The quantitative nature makes it a challenging task to improve GPC, as it is governed by several genes and affected by surrounding factors and crop management operations (Saini et al., 2020). With the genotypes, locations, and computational analysis, the heritability of GPC ranged from 0.41 to 0.70 (Giancaspro et al., 2019). Quality and quantity of protein have long been important considerations in wheat breeding. However, the negative association betwixt grain productivity and GPC, considerable environmental effects, and the narrow genetic base existing within the cultivated species of gene pool all complicate the increase in GPC (Iqbal et al., 2016). GPC improvement through traditional breeding procedures has mostly yielded mediocre results.

The combination of modern genetic tools such as DNA markers, genetic linkage maps, and high throughput phenomics platforms with genomic resources i.e. high quality wheat genome sequence and comparative genomics analysis with model species has speed up the genetic dissection of GPC in wheat cultivars (Saini et al., 2020; Gill et al., 2022). Several GPC-QTLs have been reported and located across all the chromosomes of both common wheat (e.g., Boehm et al., 2017; Krishnappa et al., 2017; Cook et al., 2018; Su et al., 2020; Jiang et al., 2021) and tetraploid wheat (Conti et al., 2011; Blanco et al., 2012; Marcotuli et al., 2017; Fatiukha et al., 2020; Ruan et al., 2021). However, very few reported QTLs could be successfully employed in molecular breeding programs mainly owing to the large confidence intervals (CIs), small phenotypic variation explained (PVE) by individual QTLs, and discrepancies in mapping results due to variations in the genetic backgrounds and environmental effects.

A meta-analysis of the QTLs identified in different experiments can be effective in refining the numbers and positions of the QTLs and detecting stable and consensus QTLs or meta-QTLs (MQTLs). It has been found that this is the most dynamic approach for the identification of genomic regions for a particular trait effectively by reducing the CI’s and therefore enhancing the detection of candidate genes (CGs) underlying the causative genomic regions (Goffinet and Gerber, 2000; Sosnowski et al., 2012; Shafi et al., 2022). Significant genomic regions are associated with several economically important traits such as grain yield and its attributing parameters (Saini et al., 2022c), nitrogen physiology (Sandhu N. et al., 2021b; Saini et al., 2021), tolerance to environmental stresses (Kumar et al., 2021; Pal et al., 2021), single disease resistance such as leaf rust and multiple disease resistance (Amo and Soriano, 2022.; Pal et al., 2022; Saini et al., 2022a) in wheat and other cereal crops. The MQTLs related to nutritional and quality traits have been reported in durum and bread wheat (Quraishi et al., 2017; Soriano et al., 2021; Gudi et al., 2022). The very first study conducted by Quraishi et al. (2017) discovered six and eight MQTLs associated with GPC and baking quality traits respectively, utilizing 155 original QTLs obtained from only eight linkage-based QTL mapping studies published before the year 2013. In the second study, Soriano et al. (2021) utilized 171 QTLs for meta-analysis and identified several MQTLs associated with different quality-related traits such as mineral contents, yellow pigment, and a few shared MQTLs for GPC in durum wheat. Most recently, Gudi et al. (2022) detected several shared MQTLs each associated with different quality traits using the studies published after the year 2013. Overall, all three above-mentioned studies utilized only a fraction of QTLs available for GPC either in durum or bread wheat; none of the studies considered all the available QTLs from both bread and durum wheat, simultaneously. Therefore, the present study was planned to integrate all the available QTLs associated with GPC in durum and bread wheat and to perform a meta-analysis for the identification of the most robust MQTLs associated with GPC.

In addition to the above, with the advancements in the next-generation sequencing (NGS) technology, high throughput genotyping strategies i.e. GBS, RAD sequencing, SNP array, and advancements in GWAS approaches, it becomes very easy to identify the significant genomic loci associated with quantitative traits in crop plants (Halder et al., 2019; Sidhu et al., 2020; Sandhu et al., 2021a; Sandhu et al., 2021b; Saini et al., 2022b). The integration of meta-analysis with GWAS has been utilized in several studies for the investigation of key genomic regions associated with economic traits (Bilgrami et al., 2020; Saini et al., 2022a; Saini et al., 2022b). The overall goal of this meta-analysis was to combine QTLs associated with GPC in tetraploid and hexaploid wheat with the aim of identification of consensus genomic regions and their confirmation through GWAS, which can be used in MQTL-assisted breeding, and to consolidate thorough information for developing novel wheat cultivars with high GPC. MQTL genes were discovered and functionally characterized. RNA-seq and microarray datasets were also used to find high-confidence CGs with significant expressions in relevant wheat tissues. The findings of this study may help in the identification of diagnostic markers and their utilization in marker-assisted breeding (MAB) or genomic selection (GS) in wheat to improve GPC.

## Materials and methods

### Collection of data on QTLs associated with grain protein content

The research articles pertaining to GPC in durum and bread wheat were collected from different repositories/databases including PubMed (https://www.pubmed.ncbi.nlm.nih.gov), Google Scholar (https://scholar.google.com/). The information on (i) markers flanking the individual QTLs (ii) peak positions and confidence intervals (CI’s) of the individual QTLs (iii) kind and size of the segregating population used in the individual studies (iv) LOD score and phenotypic variation explained (PVE) or R^2^ values were collected for each QTLs linked with GPC. Whenever the peak position of the QTLs was not given, the mid-values of the two flanking markers were used to estimate the peak positions. When there was no information on LOD scores, the threshold LOD of 3.0 was used and unique identities were assigned to individual QTLs for analysis.

**FIGURE 1:**
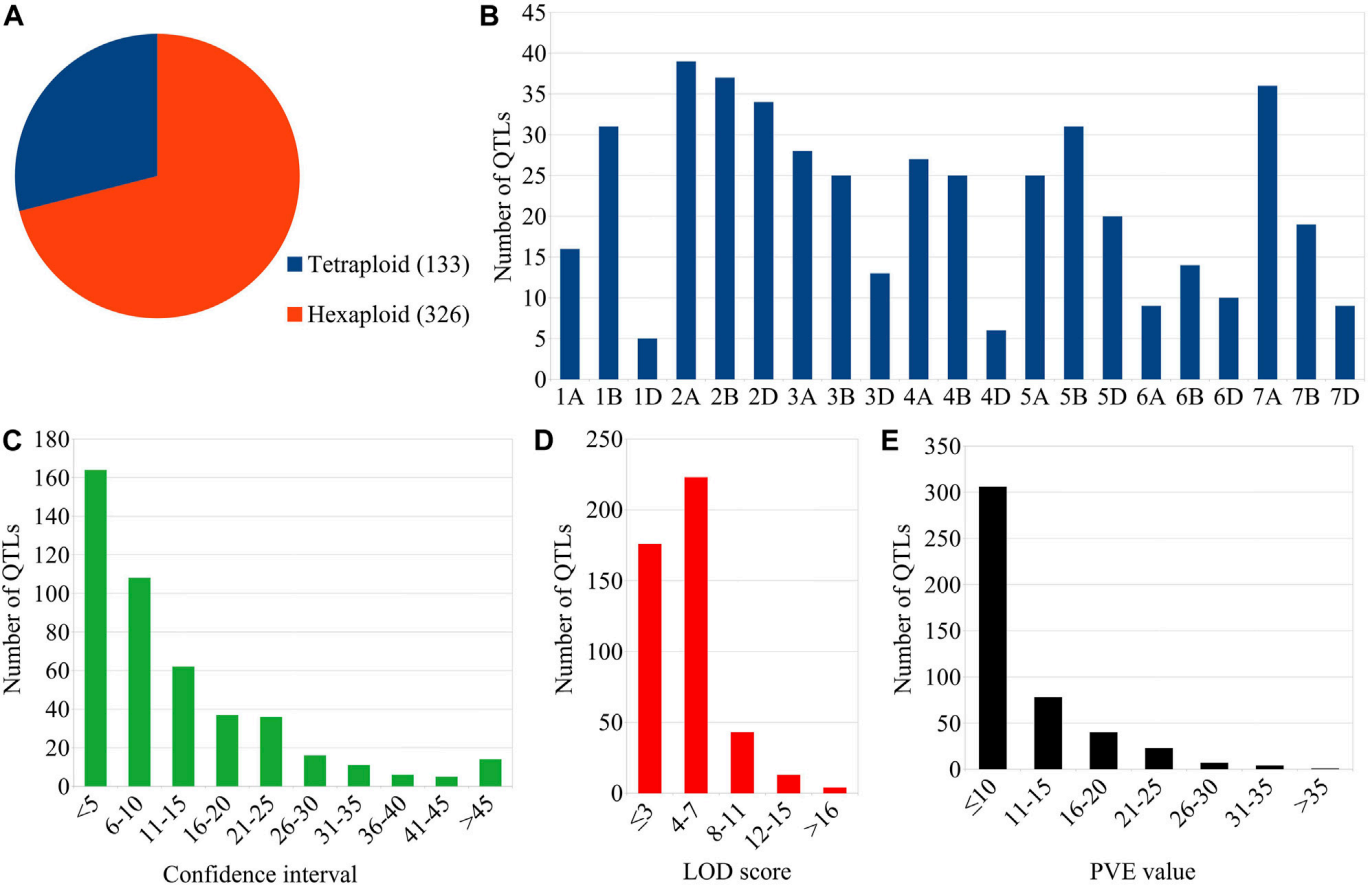
Salient features of GPC-QTLs considered during the present study. **(A)** Species-wise distribution of QTLs, **(B)** chromosome-wise distribution of QTLs, **(C)** Confidence intervals **(D)** LOD scores, and **(E)** PVE values of the QTLs.

The mapping studies utilized 49 different mapping populations (including 37 RILs, 11 DH, and one NIL population) with the size ranging from 82 to 306 (Supplementary Table S1). The size of 37 RIL populations ranged from 93 to 302, the size of 11 DH populations ranged from 95 to 414 and the size of the NIL population was 120. SSR and SNP markers were mostly utilized for mapping in these linkage-based mapping studies in wheat. As many as 459 GPC-QTLs were available from these 48 studies. Of these 459 QTLs, 133 and 326 QTLs belonged to durum and hexaploid wheat, respectively (Figure 1A).

### Construction of consensus linkage map

During present study, the markers information from previously published high-quality linkage maps used for QTL mapping in durum and common wheat for grain protein content was utilized for the development of a consensus genetic map these high-quality linkage maps are as follows- (i) the ‘Wheat, Consensus SSR, 2004’ with 1,258 marker loci (Somers et al., 2004), (ii) the ‘ITMI_SSR map’ involving 1,398 marker loci (Röder et al., 1998; Somers et al., 2004), (iii) an integrated map of durum wheat composed of 30,144 markers (Marone et al., 2013) (iv) the “Illumina iSelect 90 K SNP Array”-based genetic map with 40,267 loci (Wang et al., 2014) (v) the “AxiomR, Wheat 660 K SNP array”-based genetic map with 119,566 markers (Cui et al., 2017). The information on markers from individual investigations was used for consensus map development. The LPMerge R package was used for the development of a consensus linkage map (Endelman and Plomion, 2014). LPmerge utilizes linear programming to reduce the mean absolute error between the linkage maps and consensus maps as effectively as possible. This minimization is done under the constraints of linear inequality, which ensures that the order of the markers in the linkage maps is maintained. When linkage maps have incompatible marker orders, a minimum set of ordinal constraints is removed to resolve the problems.

### QTL projection and meta-QTL analysis

Two different files i.e., QTL file and map file were prepared from the individual QTL mapping studies. The QTL file contains the following informations: name of the QTLs, chromosomes number, linkage group, LOD scores, PVE value of individual QTLs, genetic positions of the markers flanking the QTLs, and peak positions of QTLs. Whereas, the map files mainly included the following information-population type, size, mapping function considered for mapping, chromosome-wise markers, and their respective genetic positions. These QTL files and map files were uploaded to the BioMercator V4.2 software (Sosnowski et al., 2012) and QTL projection was performed following the guidelines given in the manual (https://www.ebi.ac.uk/eccb/2014/eccb14.loria.fr/programme/id_track/ID10-summary.pdf). In the QTLs for which CI information was not available, the CI (95%) was computed from the following empirical formulas for different types of mapping populations:

CI (95%) = 530/N x R^2^ for backcross and F_2_ populations (Visscher and Goddard., 2004)
CI (95%) = 287/N x R^2^ for doubled haploid lines (Liu et al., 2009)
CI (95%) = 163/N x R^2^ for RIL lines (Guo et al., 2006)

Where N denotes the number of individuals of the concerned mapping populations utilized for mapping and R^2^ is the percentage of phenotypic variation explained (PVE) by an individual QTL. Values 530, 287, and 163 are the constants derived from simulations considering some parameters such as the proportion of recombination per cM, size of the mapping population, etc. (Visscher and Goddard, 2004; Weller and Soller, 2004; Guo et al., 2006).

The meta-QTL analysis was performed *via* the Veyrieras two-step algorithm available from the software BioMercator V4.2 for individual chromosomes. The optimal QTL model was chosen in the first step when the lowest criterion values were obtained in at least three of the five selection models [Akaike Information Criterion (AIC), Corrected AIC, AIC model-3, Bayesian Information Criterion, and Average Weight of Evidence Criterion]. In the second step, a model was used to determine the number of MQTLs on each chromosome. Finally, the consensus locations and 95% CI of the MQTLs were calculated using the variances of initial QTL positions and their intervals, respectively (Sosnowski et al., 2012).

### Determination of the physical position of the meta-QTLs

The nucleotide sequences of the MQTLs flanking markers were used for the determination of individual MQTLs physical coordinates. The flanking markers nucleotide sequences were retrieved from either of the following databases-(i) database for Triticeae and Avena (GrainGenes; https://wheat.pw.usda.gov/) for the markers such as SSR and ISSR (ii) JBrowse WHEAT UGRI (https://urgi.versailles.inra.fr/jbrowseiwgsc/) and CerealsDB for the SNP markers (https://www.cerealsdb.uk.net/cerealgenomics/CerealsDB/indexNEW.php). These sequences were BLASTed against wheat reference genome “Chinese Spring (RefSeq v1.0)” accessible at the EnsemblPlants database (https://plants.ensembl.org/index.html) to ascertain the physical positions of the markers flanking the MQTLs.

### Checking the efficacy of meta-QTLs with genome wide association study

The physical positions of significant SNPs/marker-trait associations (MTAs) related to GPC identified through 15 GWAS studies published during 2017–2022 were compared with the MQTLs genomic coordinates reported in the present study. The overlapping of MQTLs with at least one significant SNP/MTA was considered as GWAS verified MQTLs.

The 15 GWA studies involved different association panels of wheat such as spring wheat, winter wheat (hard and soft), and durum/emmer wheat. The statistic regarding the type of wheat, population size, and SNPs with GPC in wheat from different GWA studies are given in (Supplementary Table S5).

### Candidate genes and their expression analysis

The MQTLs comprising at least three initial QTLs were considered promising MQTLs which were further analyzed for candidate genes (CGs) identification. MQTLs with less than 2 Mb physical intervals were straightway examined for accessibility of gene models; whereas for the MQTLs with more than 2 Mb physical intervals, the first peak physical positions of MQTLs were estimated as per the formula used by Saini et al. (2022c) then, 2 Mb regions around the MQTL peaks were utilized for the detection of gene models. Information on genes available from each MQTL was retrieved using the BioMart tool available in the EnsemblPlants database.

Gene models detected as above were further subjected to *in silico* expression analysis using the ‘Wheat Expression Browser-expVIP’ (Expression Visualization and Integration Platform) (http://www.wheat-expression.com) (Ramírez-González et al., 2018). Relevant datasets (Gillies et al., 2012; Li et al., 2013; Pfeifer et al., 2014; Pearce et al., 2015; Clavijo et al., 2017) including expression datasets related to grains and related tissues were utilized for this purpose. Further, considering the importance of flag leaf senescence in regulating the protein contents in grains, datasets including expression data on genes showing expression during a time course of flag leaf senescence (Cantu et al., 2011; Borrill et al., 2019) were also utilized for the expression analysis. Gene models with more than 2 transcripts per million (TPM) expressions in relevant wheat tissues were considered in the current study. Further, heat maps were constructed by using the software “Morpheus” (https://software.broadinstitute.org/morpheus/) to exhibit the patterns of expressions of different genes in different tissues.

Over and above that, the nucleotide sequences of earlier associated known genes with GPC were subjected to BLAST analysis against the IWGSC RefSeq v1.0 accessible at the EnsemblePlants database. The physical coordinates of known genes were retrieved and compared with the genomic positions of MQTL regions to discover their co-localization.

## Results

### QTLs associated with grain protein content

Forty-eight (48) linkage-based mapping studies (involving 11 studies on tetraploid wheat and 37 on hexaploid wheat) pertaining to GPC-QTLs were reviewed and considered for the present study (Supplementary Table S1). The number of mapping studies, type of mapping population, and population size are described above. The chromosome-wise analysis revealed that the QTLs distribution across all the three sub-genomes was not uniform (Figure 1B). Sub-genomes A (180 QTLs) and B (182 QTLs) carried almost same number of QTLs associated with GPC, whereas, sub-genome D carried a small fraction of QTLs (only 97 QTLs). As many as 164 QTLs had a CI of less than 5 cM, whereas, the remaining QTLs had a CI of more than 5 cM with 88 QTLs possessing a CI of more than 20 cM (Figure 1C). LOD score of individual QTLs varied from ≤ 3.0 to a maximum of 31.8 with 23 QTLs having LOD scores of >10 (Figure 1D). As many as 306 QTLs contributed less than 10% variation to total phenotypic variation. There were 35 QTLs that had a PVE value of >20% (Figure 1E).

### Consensus genetic map of wheat

**FIGURE 2:**
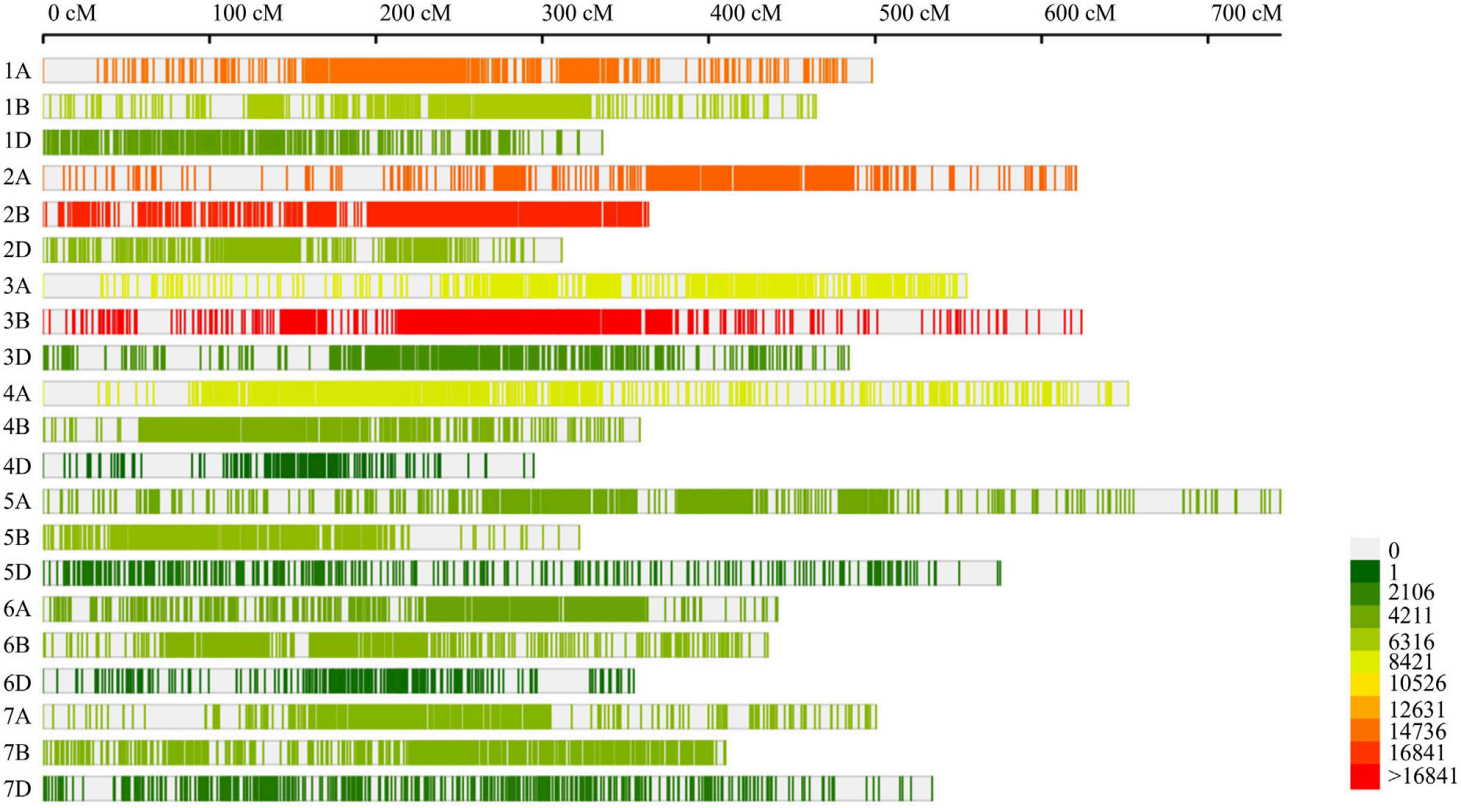
Marker density on consensus genetic map used for meta-QTL analysis in the current study. The number of loci mapped on individual chromosome.

The wheat consensus map constructed during the present study depicted significant variation for individual chromosomes with respect to genetic length (Supplementary Table S2). The consensus map covered a distance of 9,882.15 cM (chromosomal length ranging from 294.84 cM for 4D to 743.48 for 5A with an average of 470.58 cM) which accommodated 137,845 molecular markers mainly including SNPs, SSR, and other markers such as DArT, RFLP, ISSR, and AFLP. The sub-genomes A, B and D covered 4011.64, 2979.21 and 2891.3 cM genetic distances, respectively. The number of markers mapped on individual chromosomes varied from 361 markers on 4D to 18,944 markers on 3B. The sub-genome B possessed a maximum number of markers (62,780 markers) followed by subgenome A with 59,963 and subgenome D with 15,102 markers (Figure 2). The marker densities also differed among the three sub-genomes with sub-genome B showing a maximum density of 21.07 markers/cM and sub-genome D exhibiting a minimum density of 5.22 markers/cM.

### QTLs projected on the consensus map and meta-QTLs predicted for grain protein content

**FIGURE 3:**
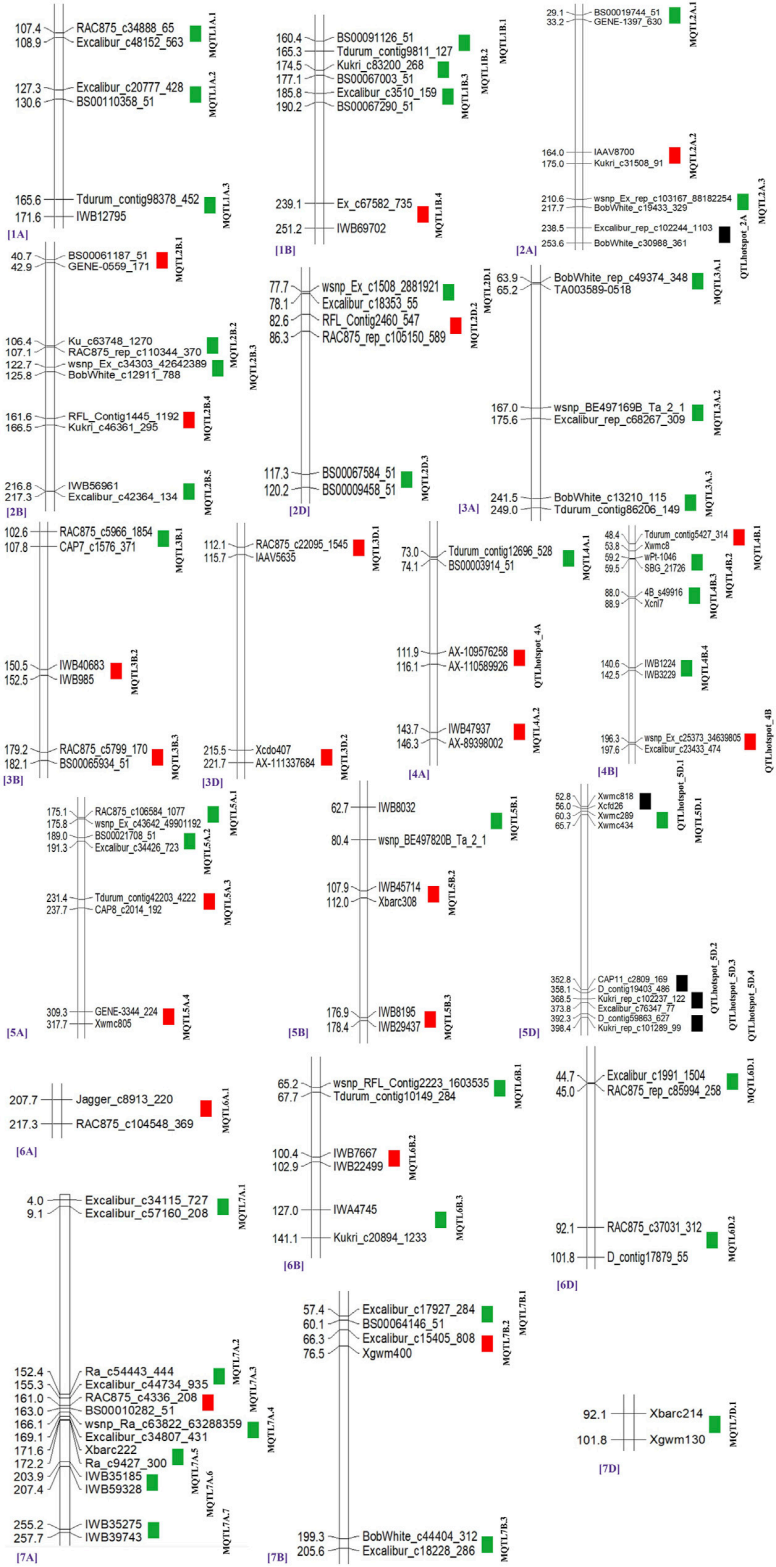
Distribution of MQTLs and QTL hotspots on different wheat chromosomes. GWAS-validated MQTL and QTL hotspots are shown with red boxes.

**FIGURE 4:**
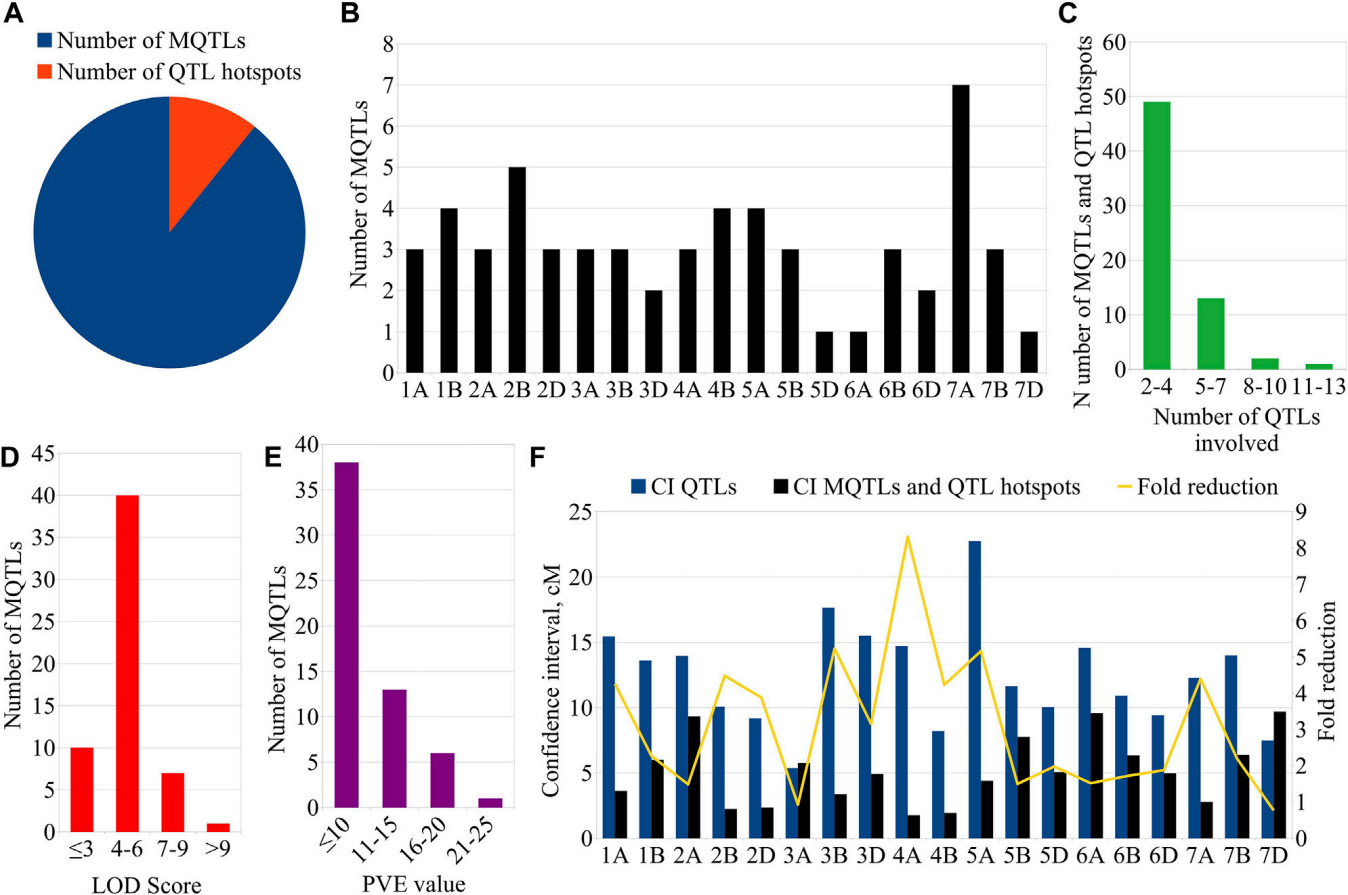
Key characteristics of MQTLs and QTL hotspots. **(A)** Proportion of MQTLs and QTL hotspots associated with GPC, **(B)** chromosome-wise distribution of MQTLs, **(C)** Number of QTLs involved in MQTLs and QTL hotspots, **(D)** LOD scores of the individual MQTLs, **(E)** PVE values of the MQTLs, **(F)** Fold reduction in CI of QTLs after meta-analysis.

From the 459 QTLs retrieved from 48 mapping studies, 304 QTLs could be projected onto the consensus genetic map. Due to some of the obvious reasons mentioned previously, the remaining 155 QTLs were unable to be projected onto the consensus map (Pal et al., 2022; Gudi et al., 2022). After QTL projection, a meta-analysis was performed which resulted in the identification of 65 potential genomic regions [including 57 MQTLs (each involving at least 2 QTLs derived from different studies) and 7 QTL hotspots (each involving multiple QTLs derived from a single study)] associated with GPC (Figures 3, 4A) based on 233 initial QTLs leaving 45 initial QTLs as singletons (single QTLs) and 26 QTLs with peaks outside the supporting intervals of identified potential genomic regions. Out of 57 MQTLs, a total of 24 MQTLs were predicted on sub-genome A, the maximum number of MQTLs was found on chromosome 7A, where there were seven MQTLs, followed by chromosome 5A which contained 4 MQTLs. In contrast, chromosomes 1A, 2A, 3A, and 4A each contained three MQTLs, and chromosome 6A had just one MQTL (Supplementary Table S3, Figure 4B).

There were 25 MQTLs available on sub-genome B, making it the sub-genome with the maximum number of MQTLs. Chromosome 2B was found to have the maximum number of 5 MQTLs. This was followed by chromosomes 1B and 4B each with 4 MQTLs, and chromosomes 3B, 5B, 6B, and 7B each with 3 MQTLs. On sub-genome D, a total of 9 MQTLs were predicted; chromosome 2D had the most, three MQTLs, followed by two MQTLs on each of chromosomes 3D, and 6D, but only one MQTL on each of chromosomes 5D and 7D, whereas, no MQTL was detected on chromosomes 1D and 4D. The number of QTLs per MQTL varied from 2 in 28 MQTLs to ≥5 QTLs in the 16 MQTLs including *MQTL7A.2* involving 10 QTLs and *MQTL3A.2* involving 13 QTLs (Figure 4C). Among the 7 QTL hotspots, 4 QTL hotspots were mapped on chromosome 5D and one each on chromosomes 2A, 4A, and 4B.

The average LOD score of the identified MQTLs varied from 2.80 (*MQTL7D.1*) to 18.40 (*MQTL6B.2*) (Supplementary Table S3, Figure 4D). The average PVE for MQTLs ranged from 3.80 (*MQTL3B.3*) to 21.34% (*MQTL7D.1*) (Figure 4E). The 57 MQTL and 7 QTL hotspots chromosome wise physical position, LOD score and PVE and CI are given (Tables 1, 2). Of the identified 57 MQTLs, nine MQTLs (viz., *MQTL2B.1*, *MQTL2D.1*, *MQTL3A.2*, *MQTL3B.1*, *MQTL4A.1*, *MQTL4A.4*, *MQTL6A.1*, *MQTL7B.2*, and *MQTL7D.1*) had more than 15% of PVE. Whereas the average PVE for QTL hotspots ranged from 6.60 to 24.78% and the number of QTLs involved in each hotspot ranged from 2 to 4 (Supplementary Table S4). With an average of 4.6 cM, the CI ranged from 0.3 to 17.71 cM for the reported MQTLs and QTL hotspots (Figure 4F). The CI reduction among the different wheat chromosomes varied significantly, with the average CI of MQTLs and QTL hotspots being 2.71 times less than that of original QTLs. The mean CI of MQTLs present on 4A reduced by 8.31 times followed by 5.23 and 5.15 times of MQTLs located on 3B and 5A, while, a slight reduction in CI was observed for MQTLs present on 3A (0.93 times) and 7D (0.77 times). The physical regions covered by MQTLs ranged from 140 bp to 224.02 Mb with an average of 15.2 Mb, whereas the physical regions occupied by QTL hotspots ranged from 1.81 Mb to 36.03 Mb with a mean of 8.82 Mb (Supplementary Table S3).

**TABLE 1:** MQTLs associated with GPC in wheat identified in the present study.

MQTL name	Chr.	Position	CI (95%)	Flanking markers	N QTLs	Avg. LOD	Avg. PVE
*MQTL1A.1*	1A	108.15	1.5	RAC875_c34888_65/Excalibur_c48152_563	3	4.50	9.23
*MQTL1A.2*	1A	128.94	3.35	Excalibur_c20777_428/BS00110358_51	7	4.34	9.78
*MQTL1A.3*	1A	168.63	6.03	Tdurum_contig98378_452/IWB12795	2	5.21	7.10
*MQTL1B.1*	1B	162.82	4.94	BS00091126_51/Tdurum_contig9811_127	5	4.80	8.44
*MQTL1B.2*	1B	175.82	2.65	Kukri_c83200_268/BS00067003_51	2	4.15	7.65
*MQTL1B.3*	1B	188.02	4.35	Excalibur_c3510_159/BS00067290_51	3	4.43	11.00
*MQTL1B.4*	1B	245.16	12.07	Ex_c67582_735/IWB69702	2	4.05	8.30
*MQTL2A.1*	2A	31.12	4.14	BS00019744_51/GENE-1397_630	7	5.90	9.77
*MQTL2A.2*	2A	169.53	11.01	IAAV8700/Kukri_c31508_91	3	3.25	6.75
*MQTL2A.3*	2A	214.17	7.14	wsnp_Ex_rep_c103167_88182254	2	5.63	7.93
*MQTL2B.1*	2B	41.78	2.17	BS00061187_51/GENE-0559_171	3	4.99	16.31
*MQTL2B.2*	2B	106.73	0.68	Ku_c63748_1270/RAC875_rep_c110344_370	3	3.07	7.23
*MQTL2B.3*	2B	124.23	3.06	wsnp_Ex_c34303_42642389/BobWhite_c12911_788	7	5.48	9.28
*MQTL2B.4*	2B	164.06	4.86	RFL_Contig1445_1192/Kukri_c46361_295	3	5.60	6.20
*MQTL2B.5*	2B	217.06	0.46	IWB56961/Excalibur_c42364_134	5	5.38	7.32
*MQTL2D.1*	2D	77.9	0.47	wsnp_Ex_c1508_2881921/Excalibur_c18353_55	7	3.53	17.55
*MQTL2D.2*	2D	84.48	3.72	RFL_Contig2460_547/RAC875_rep_c105150_589	6	5.33	10.78
*MQTL2D.3*	2D	118.76	2.93	BS00067584_51/BS00009458_51	4	6.40	7.67
*MQTL3A.1*	3A	64	1.3	BobWhite_rep_c49374_348/TA003589-0518	2	3.90	11.50
*MQTL3A.2*	3A	168.01	8.56	wsnp_BE497169B_Ta_2_1/Excalibur_rep_c68267_309	13	8.89	15.10
*MQTL3A.3*	3A	241.52	7.44	BobWhite_c13210_115/Tdurum_contig86206_149	2	3.00	9.78
*MQTL3B.1*	3B	105.2	5.26	RAC875_c5966_1854/CAP7_c1576_371	7	5.44	18.03
*MQTL3B.2*	3B	151.5	2.01	IWB40683/IWB985	4	4.63	13.48
*MQTL3B.3*	3B	180.64	2.85	RAC875_c5799_170/BS00065934_51	2	4.30	3.80
*MQTL3D.1*	3D	113.92	3.61	RAC875_c22095_1545/IAAV5635	4	4.05	10.41
*MQTL3D.2*	3D	218.61	6.23	Xcdo407/AX-111337684	4	3.82	7.34
*MQTL4A.1*	4A	73.83	0.6	Tdurum_contig12696_528/BS00003914_51	3	5.39	20.47
*MQTL4A.2*	4A	144.99	2.68	IWB47937/AX-89398002	5	4.84	15.06
*MQTL4B.1*	4B	51.09	5.48	Tdurum_contig5427_314/Xwmc8	2	6.22	9.00
*MQTL4B.2*	4B	59.32	0.3	wPt-1046/SBG_21726/IWB8981	2	6.50	8.64
*MQTL4B.3*	4B	88.44	0.83	4B_s49916/Xcnl7	6	6.82	10.13
*MQTL4B.4*	4B	141.55	1.83	IWB1224/IWB3229	2	4.65	9.91
*MQTL5A.1*	5A	175.42	0.71	RAC875_c106584_1077/wsnp_Ex_c43642_49901192	2	5.23	6.97
*MQTL5A.2*	5A	190.16	2.26	BS00021708_51/Excalibur_c34426_723	4	6.67	12.96
*MQTL5A.3*	5A	234.54	6.27	Tdurum_contig42203_4222/CAP8_c2014_192	4	3.43	7.64
*MQTL5A.4*	5A	313.52	8.4	GENE-3344_224/Xwmc805	2	4.40	10.73
*MQTL5B.1*	5B	71.52	17.71	IWB8032/wsnp_BE497820B_Ta_2_1	2	3.50	11.45
*MQTL5B.2*	5B	109.96	4.1	IWB45714/Xbarc308	7	3.66	7.09
*MQTL5B.3*	5B	177.64	1.52	IWB8195/IWB29437	2	4.10	7.55
*MQTL5D.1*	5D	62.98	5.39	Xwmc289/Xwmc434	2	3.40	8.85
*MQTL6A.1*	6A	212.49	9.58	Jagger_c8913_220/RAC875_c104548_369	2	5.94	20.00
*MQTL6B.1*	6B	66.45	2.45	wsnp_RFL_Contig2223_1603535/Tdurum_contig10149_284	5	3.02	9.98
*MQTL6B.2*	6B	101.67	2.46	IWB7667/IWB22499	2	18.40	9.65
*MQTL6B.3*	6B	134.02	14.14	IWA4745/Kukri_c20894_1233	2	3.70	4.10
*MQTL6D.1*	6D	44.81	0.3	Excalibur_c1991_1504/RAC875_rep_c85994_258	2	3.00	5.78
*MQTL6D.2*	6D	96.95	9.69	RAC875_c37031_312/D_contig17879_55	2	7.25	12.55
*MQTL7A.1*	7A	6.51	5.11	Excalibur_c34115_727/Excalibur_c57160_208	2	5.85	11.35
*MQTL7A.2*	7A	153.85	2.97	Ra_c54443_444/Excalibur_c44734_935	10	4.89	8.88
*MQTL7A.3*	7A	162	1.93	RAC875_c4336_208/BS00010282_51	2	8.07	5.01
*MQTL7A.4*	7A	167.62	3.03	wsnp_Ra_c63822_63288359/Excalibur_c34807_431	9	3.51	6.21
*MQTL7A.5*	7A	171.91	0.58	Xbarc222/Ra_c9427_300	2	8.96	11.91
*MQTL7A.6*	7A	205.68	3.48	IWB35185/IWB59328	2	2.98	7.27
*MQTL7A.7*	7A	256.45	2.45	IWB35275/IWB39743	4	6.23	4.50
*MQTL7B.1*	7B	58.74	2.64	Excalibur_c17927_284/BS00064146_51	5	6.71	4.85
*MQTL7B.2*	7B	71.42	10.22	Excalibur_c15405_808/Xgwm400/IWB36802	2	6.00	17.45
*MQTL7B.3*	7B	202.42	6.33	BobWhite_c44404_312/Excalibur_c18228_286	2	4.82	8.65
*MQTL7D.1*	7D	96.95	9.7	Xbarc214/Xgwm130	2	2.80	21.34

**TABLE 2:** QTL hotspots associated with GPC in wheat identified in the present study.

Name of QTL hotspots	Chr.	Position	CI (95%)	Flanking markers	N QTLs	Avg. LOD	Avg. PVE
*QTLhotspot_2A*	2A	246.01	15.09	Excalibur_rep_c102244_1103/BobWhite_c30988_361	2	3.50	6.60
*QTLhotspot_4A*	4A	114	4.12	AX-109576258/AX-110589926	2	3.25	7.00
*QTLhotspot_4B*	4B	196.93	1.24	wsnp_Ex_c25373_34639805/Excalibur_c23433_474	3	4.50	9.33
*QTLhotspot_5D.1*	5D	54.39	3.2	Xwmc818/Xcfd26/RAC875_rep_c72023	4	3.42	18.52
*QTLhotspot_5D.2*	5D	355.48	5.28	CAP11_c2809_169/D_contig19403_486	2	2.72	15.33
*QTLhotspot_5D.3*	5D	371.12	5.33	Kukri_rep_c102237_122/Excalibur_c76347_77	2	2.14	20.43
*QTLhotspot_5D.4*	5D	395.35	6.11	D_contig59863_627/Kukri_rep_c101289_99	2	2.75	24.78

### Verification of meta-QTLs with genome wide association study

The genomic positions of the MQTLs and QTL hotspots were compared with the genomic locations of marker-trait associations (MTAs) or significant SNPs identified in 15 earlier GWA studies (Supplementary Table S5) which utilized the association panels of hexaploid wheat (spring and winter type) and tetraploid wheat (durum and wild emmer type). This comparison enabled the identification of 19 MQTLs and 2 QTL hotspots which co-localized with 41 MTAs/SNPs identified in these previous studies (Supplementary Table S6, Figure 3). The number of MTAs/SNPs co-localized with an individual MQTL also differed. Of the 19 MQTLs, *MQTL1B.4* co-localized with a maximum of 7 MTAs/SNPs identified in 5 GWA studies (Rapp et al., 2018; Muhu-Din Ahmed et al., 2020; Jiang et al., 2021; Lou et al., 2021; Leonova et al., 2022), followed by *MQTL2B.1* co-localized with 6 MTAs/SNPs detected in 4 GWA studies (Liu et al., 2018; Chen J. et al, 2019; Liu et al., 2019; Rathan et al., 2022) and *MQTL4A.1* co-localized with 4 MTAs/SNPs identified in one GWA study (Liu et al., 2018). Three MQTLs *viz., MQTL3D.1*, *MQTL5B.3* and *MQTL7B.2* coincided with three MTAs/SNPs identified in different GWA studies.

### Candidate gene and expression analysis associated with identified meta-QTLs

**FIGURE 5:**
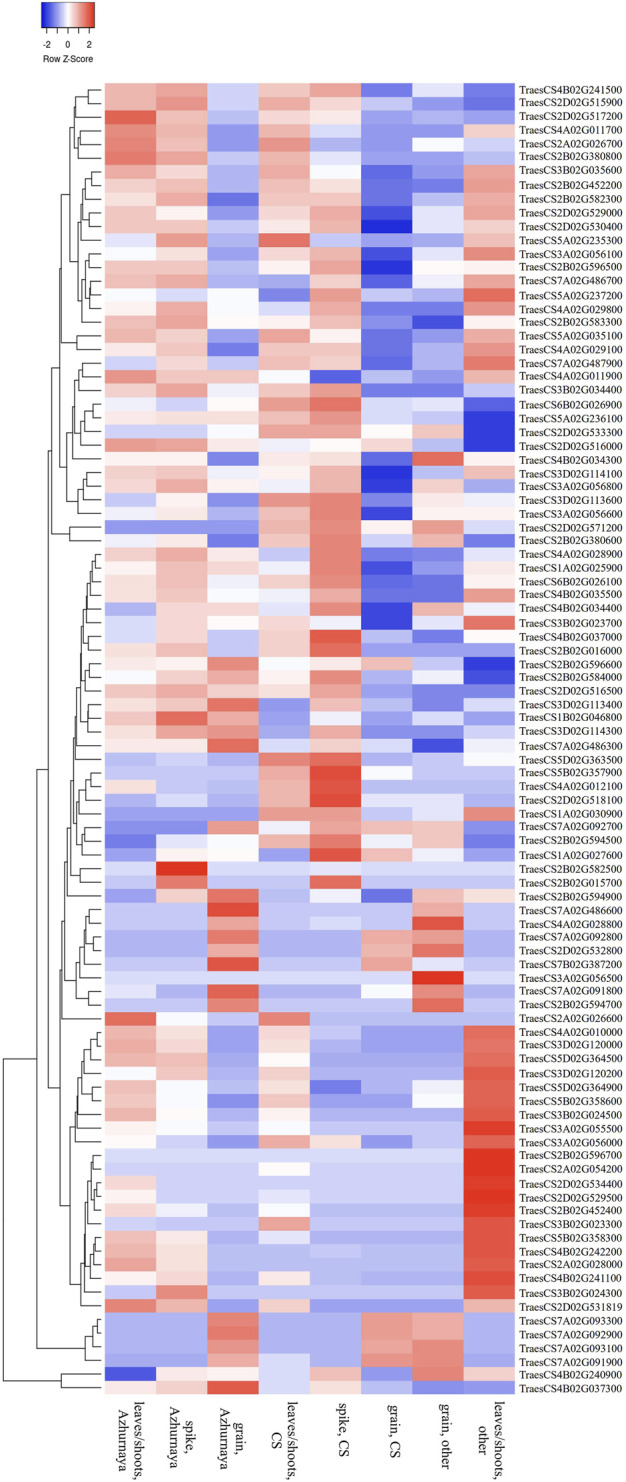
Expression patterns of selected high-confidence candidate genes in different wheat tissues.

A total of 32 promising MQTLs based on at least three original QTLs from different studies were chosen and investigated further for the identification of available gene models. This investigation enabled the identification of 705 gene models, with a maximum of 70 gene models available from *MQTL2D.2* and a minimum of only one available from *MQTL1A.2*, *MQTL1B.3*, and *MQTL7A.2* each (Supplementary Table S7). The expression analysis of 705 genes resulted in the detection of 285 significantly expressed genes with more than 2 TPM expressions in relevant wheat tissues such as leaves, spikes, and grains (Supplementary Table S7). Ninety-six promising candidate genes (CGs) believed to be associated with GPC in wheat were selected (Supplementary Table S7, Figure 5) from the significantly expressed genes which encode different types of proteins such as aminotransferases, early nodulin 93, glutamine synthetases, invertase/pectin methylesterase inhibitors, protein BIG GRAIN 1-like, cytochrome P450, Sec31, glycosyl transferases, hexokinases, small GTPases, UDP-glucuronosyl/UDP-glucosyltransferases, protein kinases, glycoside hydrolases, and EamA, SANT/Myb, GNAT, thioredoxin, phytocyanin, zinc finger, basic-leucine zipper, and homeobox domains containing proteins.

Further, a comparison of known genes for GPC with genomic regions identified through meta-analysis may also assist the efforts being made to unravel the molecular mechanisms regulating GPC in wheat. Therefore, the association of known GPC genes with MQTLs and QTL hotspots was also investigated during the present study. Five such MQTLs (viz., *MQTL1B.4*, *MQTL3A.3*, *MQTL3D.1*, *MQTL6B.3*, and *MQTL6D.2*) and 2 QTL hotspots (*QTLhotspot_4B* and *QTLhotspot_5D.4*) were found to be associated with eight genes known to regulate GPC in wheat (Supplementary Table S8). These genes include the following- *Glu-B1-1b* (encoding for HMW glutenin subunit), *Glu-1By9* (HMW glutenin subunit)*, TaNAC019-A* (endosperm-specific transcription factor), *TaNAC019-D* (endosperm-specific transcription factor), *GSr* (glutamine synthetase), *bZIP transcription factor SPA* (Basic leucine zipper TF), *GPC-B1* (NAC transcription factor), and *TaBiP1* (endoplasmic reticulum chaperone binding protein).

## Discussion

GPC is an essential trait that affects end-use quality and the economic worth of common and durum wheat (Kumar et al., 2018). Improvement in GPC content is the utmost breeding objective in wheat as chapatti making, bread making, and pasta preparation largely depend upon the GPC in both bread wheat and durum wheat. Conventional breeding techniques have been used to improve the GPC, but the expected rate of improvement has not been reached because of the strong environmental influence, the lack of a positive relationship betwixt grain yield and GPC, and the quantitative nature of the trait and low heritability (Balyan et al., 2013). With the introduction of molecular markers and next-generation sequencing, as well as other biotechnological interventions, multiple genomic regions (genes/QTLs) linked to GPC have been discovered in wheat using several mapping populations (Prasad et al., 2003; Zhao et al., 2010; Wang et al., 2012; Kumar et al., 2018; Ruan et al., 2021). Furthermore, it has been noted in several studies that QTLs found in one population may not be useful for improving traits in a different mapping population.

Meta-analysis is a novel and powerful tool which can help in integrating QTL information generated in multiple studies involving different types of populations and enable the identification of reliable and stable MQTLs linked with the target traits (Quraishi et al., 2017). Meta-analyses for different traits have been reported in major food crops such as rice, wheat, maize, etc., (Quraishi et al., 2017; Hu et al., 2021; Prakash et al., 2022). In wheat, meta-analyses have been performed for several traits which include yield and yield-associated parameters (Saini et al., 2022c), quality traits (Quraishi et al., 2011; Shariatipour et al., 2021; Soriano et al., 2021; Singh et al., 2022; Gudi et al., 2022); disease resistance (Liu et al., 2009; Soriano and Royo, 2015; Venske et al., 2019; Liu et al., 2020; Jan et al., 2021; Saini et al., 2022a) and abiotic stress tolerance (Kumar et al., 2021; Pal et al., 2021; Soriano et al., 2021). Previously, meta-analyses of QTLs linked with quality attributes in wheat were also undertaken (Quraishi et al., 2017; Soriano et al., 2021; Gudi et al., 2022).

Quraishi et al. (2017) found six MQTLs for GPC and eight MQTLs for baking quality utilizing only 155 QTLs in hexaploid wheat for the first time. Recently in 2021, Soriano et al. utilized 171 QTLs associated with different quality traits (viz., arabinoxylan, β-glucan, flour yellow color, grain mineral contents, GPC, SDS-sedimentation volume, and yellow pigment content) and identified 17 shared MQTLs (including QTLs for different quality traits, biotic and abiotic stress parameters) in durum wheat (Soriano et al., 2021). Most recently in 2022, Gudi et al. utilized QTLs reported after the year 2013 and identified several shared MQTLs for GPC (co-localized with different processing quality traits, dough rheology attributes, and nutritional traits). These earlier studies primarily involved the prediction of MQTLs for different traits taken together for different quality traits, biotic and abiotic stress parameters, and no effort was made to identify MQTLs for GPC utilizing all the available QTLs from both bread and durum wheat, thus reducing their utility in wheat breeding.

In contrast, the present study includes the projection of 304 QTLs out of 459 QTLs collected from literature published to date for GPC. The proportion of GPC QTLs projected on the consensus map in the current study is much greater than in earlier studies (Quraishi et al., 2017; Soriano et al., 2021; Gudi et al., 2022), which could be attributed to the presence of many QTLs and the use of a highly dense consensus map in the current study. In the present study, 57 MQTLs and 7 QTL hotspots associated with GPC were identified which were distributed across the three sub-genomes. The detection of 57 MQTLs and 7 QTL hotspots from 304 QTLs resulted in a 4.68-fold (304/65) reduction in the number of QTLs or genomic regions linked with GPC in wheat. Physical positions of four MQTLs (*MQTL1B.4*, *MQTL4B.3*, *MQTL7B.2*, and *MQTL7B.3*) predicted during the current study were reported to be overlapped with four MQTLs (durum*MQTL1B.3*, durum*MQTL4B.4*, durum*MQTL7B.1*, and durum*MQTL7B.9*) earlier identified to be associated with GPC in durum wheat (Soriano et al., 2021).

From a breeding viewpoint, it is important to determine the most reliable and robust MQTLs each based on numerous initial QTLs found in the various populations and environments. In the current study, 16 MQTLs each based on more than 5 original QTLs were observed. There were up to 13 initial QTLs associated with GPC in one MQTL on chromosome 3A (M*QTL3A.2*), which is significantly more than what was reported in earlier meta-analyses (Quraishi et al., 2017; Soriano et al., 2021; Gudi et al., 2022). The present study compiled extensive data on QTLs from different mapping populations. It effectively reduced the QTLs' CIs, enhancing the reliability of CG detection from potential MQTL regions. The mean CIs of MQTLs were 2.71 times lower than the CIs of the original QTLs included in the meta-analysis. As many as 15 MQTLs had CIs of less than 2 cM.

### Validating meta-QTLs/QTL hotspots with genome wide association study

GWAS is an efficient approach for the dissection of complex traits by utilizing natural genetic diversity (Korte and Farlow, 2013). It is based on the principle of linkage disequilibrium which provides high-resolution power and allows the identification of significant MTAs or SNPs by utilizing high throughput genotyping and precise phenotypic data (Gupta et al., 2005). Meta-QTL analysis and GWAS both have their advantages and limitations that can complement each other. There were significant overlaps between the MQTLs predicted in this study and the MTAs identified by GWAS for GPC in wheat. Out of the 57 predicted MQTLs and 7 QTL hotspots, 19 MQTLs and 2 QTL hotspots overlapped with MTAs identified for GPC in recent GWA studies in wheat. In some of the earlier studies of meta-analysis, MQTLs for other traits of economic importance have also been validated using this method (Aduragbemi and Soriano, 2021; Gudi et al., 2022; Saini et al., 2021; Pal et al., 2022; Yang et al., 2021). In these earlier studies, only 38.66, 47.22, 78.57, 58.33, 69.23, and 61.37% of the physically anchored MQTLs were confirmed using GWAS data.

The number of MQTLs found in the current study that was confirmed by GWAS is within the range of MQTLs found in earlier studies. The varying proportions of MQTLs validated by GWAS-based MTAs/SNPs in different studies may be due to either of the following reasons: (i) the genetic material utilized in interval mapping (eventually in meta-analysis) and GWAS was completely different, (ii) neither method fully accounted for the genetic variations present in the gene pool for the target trait(s), (ii) GWAS is intended to detect MTAs with a minor allele frequency of more than 5%; nevertheless, linkage-based mapping studies can uncover rare alleles with more severe phenotypic effects, (iv) there were varying number of GWAS-MTAs available for analysis, (v) accuracy of physical positions of MQTLs to be compared with MTAs.

### MQTL-assisted breeding for grain protein content improvement in wheat

Individual MQTL LOD scores varied from 2.80 to 18.40, with a mean of 5.15, whereas PVE values ranged from 3.80 to 21.34 percent, with a mean of 10.14 percent. Based on the above findings, the MQTLs were further filtered to identify some of the promising MQTLs for breeding, which we termed breeders' MQTLs, based on the following criteria: (a) CI less than 2.5 cM, (ii) PVE more than 10%, (iii) LOD more than 3.5, and (iv) dependency on at least three original QTLs from multiple studies; this effort enabled the detection of six breeder’s MQTLs (*viz.*, *MQTL2B.1*, *MQTL2D.1*, *MQTL3B.2*, *MQTL4A.1*, *MQTL4B.3*, and *MQTL5A.2*) each located on different chromosomes 2B, 2D, 3B, 4A, 4B, and 5A (Supplementary Table S9). Three of these MQTLs (viz., *MQTL2B.1*, *MQTL3B.2*, *MQTL4A.1*) were also validated by GWA studies. The selected breeder’s MQTLs could be effectively utilized in MQTL-assisted breeding for the genetic enhancement of GPC in wheat. Two other MQTLs (viz., *MQTL3A.2* and *MQTL3B.1*) located on chromosomes 3A and 3B, respectively, explained more than 15% of the phenotypic variations but had large CIs (8.56 and 5.26 cM, respectively) making them unsuitable for breeding programs. Although, these MQTLs could be considered for fine mapping and cloning in future studies.

### Candidate genes associated with grain protein content

In the present study, a total of 705 gene models available from 32 promising MQTL regions were detected. Out of these 705 gene models, as many as 285 gene models (available from 30 MQTLs) showed significant expressions in different wheat tissues. The *MQTL2D.2* had the maximum number of 35 significantly expressed genes, whereas, *MQTL1B.3*, *MQTL7A.2* had no significantly expressed gene. Among the 285 significantly expressed genes, 96 high-confidence CGs were selected based on their probable roles in the regulation of GPC in wheat (Table 3). These genes encode for different proteins such as follows-aminotransferases, early nodulin 93, invertase/pectin methylesterase inhibitors, protein BIG GRAIN 1-like, cytochrome P450, glycosyl transferases, hexokinases, small GTPases, UDP-glucuronosyl/UDP-glucosyltransferases, and EamA, CBS, SANT/Myb, GNAT, thioredoxin, phytocyanin, and homeobox domains containing proteins. In an earlier study, Quraishi et al. (2017) identified three genes *Triticin*, *Gliadin*, *Tri-ribulose-1,5-bisphosphate carboxylase*/*Viviparous* as the candidates for three MQTLs located on chromosomes 1A, 2A, and 3A. Most recently in the year 2022, Gudi et al. identified 44 CGs for different quality traits in wheat. The majority of these genes were linked to proteins that bind metal ions, Zn-transporters, small hydrophilic seed proteins, amino acid transporters, sweet-sugar transporters, UDP-glucuronosyl/UDP-glucosyltransferases, sugar/inositol transporters, and other proteins (Gudi et al., 2022).

**TABLE 3:** High-confidence candidate genes associated with GPC in wheat.

MQTL	Gene ID	Start (bp)	End (bp)	Function description
*MQTL1A.1*	TraesCS1A02G025900	12363666	12369892	Protein kinase domain
*MQTL1A.1*	TraesCS1A02G027600	13081413	13086602	Elongation factor EFG, domain V-like
*MQTL1A.2*	TraesCS1A02G030900	14253391	14257167	Protein kinase domain
*MQTL1B.1*	TraesCS1B02G046800	26621671	26638285	F-box associated domain, type 3
*MQTL2A.1*	TraesCS2A02G026600	12287484	12289174	NADH:flavin oxidoreductase/NADH oxidase, N-terminal
*MQTL2A.1*	TraesCS2A02G026700	12289478	12291551	Oxoglutarate/iron-dependent dioxygenase
*MQTL2A.1*	TraesCS2A02G028000	12911511	12913256	UDP-glucuronosyl/UDP-glucosyltransferase
*MQTL2A.2*	TraesCS2A02G054200	22571240	22573616	UDP-glucuronosyl/UDP-glucosyltransferase
*MQTL2B.1*	TraesCS2B02G015700	7565607	7579969	Cytochrome P450
*MQTL2B.1*	TraesCS2B02G016000	7645446	7648835	Hexokinase
*MQTL2B.2*	TraesCS2B02G583300	770679795	770687184	Clathrin, heavy chain/VPS, 7-fold repeat
*MQTL2B.2*	TraesCS2B02G582300	769949352	769950767	Signal transduction response regulator, receiver domain
*MQTL2B.2*	TraesCS2B02G584000	771184907	771188118	Pentatricopeptide repeat
*MQTL2B.2*	TraesCS2B02G582500	770055312	770056235	Phytocyanin domain
*MQTL2B.3*	TraesCS2B02G594500	779101022	779102772	TRAF-like
*MQTL2B.3*	TraesCS2B02G594700	779179160	779184177	Glycoside hydrolase, family 32
*MQTL2B.3*	TraesCS2B02G594900	779251292	779256095	Glycoside hydrolase, family 32
*MQTL2B.3*	TraesCS2B02G596600	779861522	779864156	Zinc finger, RING-type
*MQTL2B.3*	TraesCS2B02G596500	779854577	779858004	GHMP kinase, ATP-binding, conserved site
*MQTL2B.3*	TraesCS2B02G596700	779880390	779882468	Protein kinase domain
*MQTL2B.4*	TraesCS2B02G452400	646411925	646417556	Protein kinase domain
*MQTL2B.4*	TraesCS2B02G452200	646210092	646214150	Glycosyl transferase, family 14
*MQTL2B.5*	TraesCS2B02G380600	544981351	544984884	GDSL lipase/esterase
*MQTL2B.5*	TraesCS2B02G380800	544990354	544996445	WD40 repeat
*MQTL2D.1*	TraesCS2D02G518100	608537487	608541232	EamA domain
*MQTL2D.1*	TraesCS2D02G516000	607418412	607425112	Zinc finger, RING-type
*MQTL2D.1*	TraesCS2D02G515900	607283456	607284936	AP2/ERF domain
*MQTL2D.1*	TraesCS2D02G517200	608196430	608200931	Carbohydrate kinase, FGGY
*MQTL2D.1*	TraesCS2D02G516500	607933268	607938429	Zinc finger, UBP-type
*MQTL2D.2*	TraesCS2D02G533300	617965028	617967864	Zinc finger, CCHC-type
*MQTL2D.2*	TraesCS2D02G530400	616952435	616955208	Glycosyl transferase, family 8
*MQTL2D.2*	TraesCS2D02G529500	616625519	616627949	Zinc finger, RING-type
*MQTL2D.2*	TraesCS2D02G532800	617894612	617897359	Glycosyltransferase 2-like
*MQTL2D.2*	TraesCS2D02G534400	618138520	618143248	Protein kinase domain
*MQTL2D.2*	TraesCS2D02G529000	616525223	616530794	Basic-leucine zipper domain
*MQTL2D.2*	TraesCS2D02G531819	617414356	617417584	Pentatricopeptide repeat
*MQTL2D.3*	TraesCS2D02G571200	637649854	637652332	Sugar phosphate transporter domain
*MQTL3A.2*	TraesCS3A02G055500	32145925	32149547	Glycosyl transferase, family 1
*MQTL3A.2*	TraesCS3A02G056000	32251515	32253338	SANT/Myb domain
*MQTL3A.2*	TraesCS3A02G056100	32384710	32387330	SANT/Myb domain
*MQTL3A.2*	TraesCS3A02G056500	32639616	32642710	Small GTPase
*MQTL3A.2*	TraesCS3A02G056600	32646665	32650869	Small GTPase
*MQTL3A.2*	TraesCS3A02G056800	32716216	32718252	Small GTPase
*MQTL3B.1*	TraesCS3B02G023300	10013879	10014906	Zinc finger, RING-type
*MQTL3B.1*	TraesCS3B02G023700	10198656	10202090	Glycosyltransferase 61
*MQTL3B.1*	TraesCS3B02G024300	10388175	10392856	Protein kinase domain
*MQTL3B.1*	TraesCS3B02G024500	10562122	10573523	Protein kinase domain
*MQTL3B.2*	TraesCS3B02G034400	16439668	16444830	WD40 repeat
*MQTL3B.2*	TraesCS3B02G035600	17558579	17563046	SUF system FeS cluster assembly, SufBD
*MQTL3D.1*	TraesCS3D02G120000	75733615	75740231	Serine incorporator/TMS membrane protein
*MQTL3D.1*	TraesCS3D02G120200	75946541	75948546	UDP-glucuronosyl/UDP-glucosyltransferase
*MQTL3D.2*	TraesCS3D02G113400	67470659	67484580	WD40 repeat
*MQTL3D.2*	TraesCS3D02G113600	67541388	67544595	Transferase
*MQTL3D.2*	TraesCS3D02G114100	67710273	67713074	Phosducin, thioredoxin-like domain
*MQTL3D.2*	TraesCS3D02G114300	67717667	67729719	Homeobox domain
*MQTL4A.2*	TraesCS4A02G010000	5835053	5839914	Protein kinase domain
*MQTL4A.2*	TraesCS4A02G011700	6801496	6803203	PsbQ-like domain superfamily
*MQTL4A.2*	TraesCS4A02G011900	6884841	6888795	GNAT domain
*MQTL4A.2*	TraesCS4A02G012100	6921978	6923456	Protein BIG GRAIN 1-like
*MQTL4A.4*	TraesCS4A02G028800	21057927	21058645	Phytocyanin domain
*MQTL4A.4*	TraesCS4A02G028900	21062686	21066407	Myc-type, basic helix-loop-helix (bHLH) domain
*MQTL4A.4*	TraesCS4A02G029100	21215068	21220471	SLC26A/SulP transporter
*MQTL4A.4*	TraesCS4A02G029800	21845172	21854022	Protein kinase domain
*MQTL4B.3*	TraesCS4B02G034300	25263926	25266948	Ribosomal protein S21
*MQTL4B.3*	TraesCS4B02G034400	25267031	25270444	Ribosomal protein L18
*MQTL4B.3*	TraesCS4B02G035500	25842359	25852716	CBS domain
*MQTL4B.3*	TraesCS4B02G037000	26791996	26794269	Zinc finger, CCCH-type
*MQTL4B.3*	TraesCS4B02G037300	27104920	27110487	BRCT domain
*MQTL4B.5*	TraesCS4B02G241100	500052749	500054910	Cytochrome P450
*MQTL4B.5*	TraesCS4B02G241500	500252893	500257371	Protein kinase domain
*MQTL4B.5*	TraesCS4B02G242200	500868125	500871590	Protein kinase domain
*MQTL4B.5*	TraesCS4B02G240900	499898695	499901767	Glutamine synthetase, catalytic domain
*MQTL5A.2*	TraesCS5A02G035100	32702327	32704442	Methyltransferase type 11
*MQTL5A.3*	TraesCS5A02G235300	451454753	451458817	Glycosyl transferase, family 31
*MQTL5A.3*	TraesCS5A02G236100	451731021	451736139	Aminotransferase, class I/classII
*MQTL5A.3*	TraesCS5A02G237200	452933936	452937774	Basic-leucine zipper domain
*MQTL5B.2*	TraesCS5B02G358300	537960131	537964109	Cytochrome P450
*MQTL5B.2*	TraesCS5B02G358600	538541865	538548795	Zinc finger C2H2-type
*MQTL5B.2*	TraesCS5B02G357900	537530437	537537053	F-box-like domain superfamily
*MQTL5D.1*	TraesCS5D02G364500	441919689	441927139	Cytochrome P450
*MQTL5D.1*	TraesCS5D02G364900	442330660	442339062	Zinc finger C2H2-type
*MQTL5D.1*	TraesCS5D02G363500	441327117	441328139	Sulfotransferase domain
*MQTL6B.1*	TraesCS6B02G026100	15780439	15788214	Ancestral coatomer element 1, Sec16/Sec31
*MQTL6B.1*	TraesCS6B02G026900	15929467	15933792	Aspartate/other aminotransferase
*MQTL7A.4*	TraesCS7A02G091800	55918294	55919130	Early nodulin 93 ENOD93 protein
*MQTL7A.4*	TraesCS7A02G091900	55922722	55923573	Early nodulin 93 ENOD93 protein
*MQTL7A.4*	TraesCS7A02G092700	56244809	56245904	Early nodulin 93 ENOD93 protein
*MQTL7A.4*	TraesCS7A02G092800	56324077	56324854	Early nodulin 93 ENOD93 protein
*MQTL7A.4*	TraesCS7A02G092900	56430737	56431572	Early nodulin 93 ENOD93 protein
*MQTL7A.4*	TraesCS7A02G093100	56658022	56658811	Early nodulin 93 ENOD93 protein
*MQTL7A.4*	TraesCS7A02G093300	56738537	56739378	Early nodulin 93 ENOD93 protein
*MQTL7A.7*	TraesCS7A02G486300	676584400	676591905	Thioredoxin domain
*MQTL7A.7*	TraesCS7A02G486600	677692146	677692709	Invertase/pectin methylesterase inhibitor domain superfamily
*MQTL7A.7*	TraesCS7A02G486700	677694300	677697668	Zinc finger, RING-type
*MQTL7A.7*	TraesCS7A02G487900	678152379	678157242	Protein kinase domain
*MQTL7B.1*	TraesCS7B02G387200	653168095	653169641	Aspartic peptidase domain superfamily

The association of these high-confidence CGs with GPC may be discussed as follows- (i) Protein accumulation during the grain-filling stage is aided by the remobilization of amino acids from vegetative tissues, a procedure that is predicted to involve both amino acid importers and exporters. In a recent study in wheat, the UMAMIT family of transporters was characterized, with the majority of them carrying EamA domains. Gene *TaUMAMIT17* exhibited significant amino acid export activity and played a key role in the enhancement of GPC (Fang et al., 2022). (ii) In a more recent study, semi-dominant alleles for a class III homeodomain-leucine zipper TF, *HOMEOBOX DOMAIN-2* (*HB-2*) were identified which generate more flower-bearing spikelets and significantly improve GPC. (iii) Aminotransferases are known to enhance root absorption of a range of amino acids and to affect GPC positively (Peng et al., 2014). (iv) The endoplasmic reticulum produces the seed storage proteins glutelin and beta-globulin, which are then put into protein storage vacuoles. Small GTPase Sar1, which transports secretory proteins from the endoplasmic reticulum to the Golgi apparatus, is known to act as a molecular switch to regulate the assembly of coat protein complex II (Tian et al., 2013). (v) CBS domain-containing proteins are believed to have regulatory functions; therefore, such proteins may be functional in improving GPC in wheat grains (Leonova et al., 2022). (vi) Secretory24 (Sec24) and Sec31 (Sec31) promote anterograde transport of newly generated proteins from the endoplasmic reticulum to distinct compartments in the plant endometrium through shell protein complex II (Lv et al., 2021). (vii) Glutamine synthetases are known to play key roles in plant nitrogen assimilation and ammonium detoxification thereby regulating GPC in durum wheat (Nigro et al., 2016). (viii) Several members of the basic leucine zipper (bZIP) family have been identified to play a key role in the regulation of wheat grain storage protein synthesis (Li et al., 2020; Pfeifer et al., 2014). (ix) ENOD93 encodes early nodulin 93 proteins which are known to regulate nitrogen use efficiency in different crops including rice and wheat (Kant et al., 2010; Saini et al., 2021), thereby believed to play key roles in the regulation of GPC in wheat grains. Some of the key CGs discovered in this study may be validated or functionally characterized utilizing various methods such as over-expression, genome editing, knockout techniques, etc.

Comparing genomic regions identified through meta-analysis to known GPC genes can assist researchers in better comprehending the genetic architecture underpinning GPC. As a result, during the current study, a connection of MQTLs with known GPC genes was also explored. This study detected the co-localization of eight functionally known GPC genes with different MQTL regions, including *Glu-B1-1b* (Ravel et al., 2006), *Glu-1By9* (Chen J. et al., 2019), *GPC-B1* (Uauy et al., 2006), *TaBiP1* (Zhu et al., 2014), *GSr* (Bernard et al., 2008), *TaNAC019-A* (Gao et al., 2021), *TaNAC019-D* (Gao et al., 2021), and *bZIP-TF SPA* (Boudet et al., 2019). *MQTL1B.4* contained the *Glu-B1-1b* and *Glu-1By9* genes, which are precursors of high-molecular-weight glutenin subunits, which produce glutenin when combined with low-molecular-weight subunits. Gluten proteins, which account for over 80% of total GPC, are produced by roughly the same amount of glutenins and gliadin (Ravel et al., 2006; Chen Q. et al., 2019). *MQTL6B.3* contained the major gene *GPC-B1* which encodes a NAC TF that causes 10–15% increase in GPC in wheat (Uauy et al., 2006). *TaBiP1*, co-localized with *MQTL6D.2*, encodes an important functional protein i.e., endoplasmic reticulum chaperone binding protein which is involved in the bio-synthesis of subunit types of high molecular weight-glutenin subunit (Zhu et al., 2014). *GSr*, which is co-localized with *QTLhotspot_4B*, encodes glutamine synthetase, which is important in absorbing ammonia at the key stages of nitrogen remobilization to the grain, hence regulating the GPC in wheat grains (Bernard et al., 2008). *TaNAC019-A* and *TaNAC019-D*, available from *MQTL3A.3* and *MQTL3D.1*, respectively, encode NAC TFs that regulate starch and glutenin accumulation and its elite allele increases grain quality in wheat (Gao et al., 2021). bZIP transcription factor SPA, co-localizing with *QTLhotspot_5D.4*, is known to repress glutenin synthesis in common wheat (Boudet et al., 2019).

## Conclusion

The current work is the first thorough meta-analysis of GPC QTLs in common and durum wheat. The meta-analysis identified 57 MQTLs and 7 QTL hotspots associated with GPC, of which 19 MQTLs and 2 QTL hotspots were also validated with GWA studies. Within these MQTL regions, 705 gene models were detected; of these genes, 285 genes displayed significant expression across different wheat tissues analyzed; and 96 high-confidence genes were chosen based on functional annotation, expression analysis, and literature survey and proposed for future basic studies. Additionally, data on the markers flanking the MQTLs can be included in genomic selection models to increase the precision of GPC predictions in wheat. Wheat breeders may make greater use of selected breeder’s MQTLs (viz., *MQTL2B.1*, *MQTL2D.1*, *MQTL3B.2*, *MQTL4A.1*, *MQTL4B.3*, and *MQTL5A.2*) and CGs uncovered in this study for genetic improvement of GPC in wheat.

## Data Availability

The datasets presented in this study can be found in online repositories. The names of the repository/repositories and accession number(s) can be found in the article/Supplementary Material and data on QTLs associated with GPC have also been made publicly available through a most comprehensive wheat QTL database (http://wheatqtldb.net/).

## Abbreviations

CG, candidate gene; GWAS, genome wide association study; GPC, grain protein content; MQTL, Meta QTL.
